# Kinesthetic Coordination Abilities in 6-Year-Old Children: School Quintile, Gender, and Hand Dominance Differences

**DOI:** 10.1007/s13158-023-00350-5

**Published:** 2023-02-18

**Authors:** Carmen Bonafede, Elna van der Merwe

**Affiliations:** Department of Exercise & Sport Sciences, School of Health and Rehabilitation Sciences, Faculty of Health Sciences, University of the Free State, 205 Nelson Mandela Drive, Park West, Bloemfontein, 9301 Free State South Africa

**Keywords:** Handedness, Kinaesthesia, Gender, Socio-economic status, Proprioception

## Abstract

Proprioceptive kinaesthetic control underpins motor movements of developing children and can be influenced by several factors. The main aim of this study was to establish proprioceptive kinaesthetic coordination differences in six-year-olds from different school quintiles, of different genders, and with different handedness. A total of 193 six-year-olds from 10 schools of different quintiles in the Motheo District, Mangaung, were included, of which 97 (50.3%) were boys and 96 (49.7%) were girls. A quantitative cross-sectional study design was followed to determine proprioceptive kinaesthetic coordination differences. Right-handed participants performed significantly better than left-handed participants in the Finger-to-Nose task (*p* = 0.0125) when moving and positioning their dominant arm and hand. When using their dominant arm, significant differences in the shoulder-level-arm-raise (*p* = 0.0288) favoured boys. Girls showed superior execution of the force perception task (*p* = 0.0322). In conclusion, significant proprioceptive kinaesthetic coordination differences in six-year-olds were mainly not evident. Future work should explore proprioceptive kinaesthetic coordination differences in children of other ages and determine the practical implications of identified differences.

## Résumé

Le contrôle kinesthésique proprioceptif sous-tend les mouvements moteurs des enfants en pleine croissance et peut être influencé par plusieurs facteurs. Cette étude visait principalement à identifier les différences dans la coordination kinesthésique proprioceptive chez les enfants de six ans de différents quintiles d’école, de différents sexes, et avec différentes manualités. Au total, 193 enfants de six ans provenant de 10 écoles de quintiles différents du district de Motheo, à Mangaung, ont été inclus, dont 97 (50,3 %) étaient des garçons et 96 (49,7 %) des filles. Une étude transversale quantitative a été menée pour déterminer les différences dans la coordination kinesthésique proprioceptive. En ce qui concerne l’épreuve doigt-nez, les participants droitiers ont obtenu de bien meilleurs résultats que les participants gauchers (p = 0,0125) lors du déplacement et du positionnement de leur bras et leur main dominants. Lors de l’utilisation de leur bras dominant, des différences significatives dans l’épreuve de levée de bras jusqu’aux épaules (p = 0,0288) favorisaient les garçons. Les filles ont démontré une meilleure exécution lors de l’épreuve de perception de la force (p = 0,0322). En conclusion, les différences significatives dans la coordination kinesthésique proprioceptive chez les enfants de six ans n’étaient généralement pas évidentes. Les travaux futurs devraient explorer les différences dans la coordination kinesthésique proprioceptive chez les enfants d’autres âges et déterminer les implications pratiques des différences identifiées.

## Resumen

El control propioceptivo cinestésico sustenta los movimientos motores de los niños en desarrollo y puede estar influido por diversos factores. El objetivo principal de este estudio fue establecer las diferencias de coordinación propioceptiva cinestésica en niños de seis años de edad de diferentes grupos escolares, de diferentes géneros y con diferente lateralidad. Se incluyeron 193 niños de seis años de 10 escuelas de diferentes grupos del distrito de Motheo, Mangaung, de los cuales 97 (50,3%) eran niños y 96 (49,7%) niñas. Se siguió un diseño de estudio cuantitativo transversal para determinar las diferencias de coordinación propioceptiva cinestésica. Los participantes diestros rindieron significativamente mejor que los zurdos en la tarea Dedo a Nariz (p = 0,0125) cuando movían y colocaban su brazo y mano dominantes. Cuando utilizaban su brazo dominante, las diferencias significativas en la elevación del hombro-brazo (p = 0,0288) favorecían a los chicos. Las chicas mostraron una ejecución superior en la tarea de percepción de la fuerza (p = 0,0322). En conclusión, las diferencias significativas en la coordinación propioceptiva cinestésica en niños de seis años no fueron evidentes en su mayoría. Futuros trabajos deberían explorar las diferencias de coordinación propioceptiva cinestésica en niños de otras edades y determinar las implicaciones prácticas de las diferencias identificadas.

## Introduction

Kinaesthesia and proprioception are said to be synonymous (Han et al., 2016) as both strongly refer to internal sensory information enabling individuals to be aware of the position and movement of their body and limbs. Kinaesthetic sense is derived from the motor commands which generate a motor movement (Proske, 2006) and can be defined as the awareness of limb position and movements provided by the joint receptors (Proske, 2006; Proske & Gandevia, 2012; Siegel & Sapru, 2011). Proprioception can be seen as position sense and movement of one’s own body parts, as well as tension sense or force perception, together with the effort exerted by relevant muscles (Bornstein et al., 2021). Consequently, the proprioceptive system enables children to make movement corrections as they move (Magill & Anderson, 2017), while input from cutaneous receptors and muscle receptors possibly assists kinaesthesia at joints throughout the body (Collins et al., 2005).

Intact kinaesthesia is necessary for effective motor performance and postural control (Li et al., 2015), while proprioception—especially in the initial stage of motor development (two-six years)—has been established as a central aspect for movement control and motor learning (Úbeda-Pastor et al., 2018). Úbeda-Pastor and co-workers (2018) reported that proprioception greatly affects children’s postural and balancing skills by the age of seven. As seen from the definitions and reported findings, kinaesthetic sense and proprioception play an important role in controlling and coordinating motor movements. For the purpose of this study, the term *proprioceptive kinaesthetic coordination* will be used as a collective, referring to the involvement of kinaesthesia and proprioception to bring about motor movements which are controlled and well coordinated.

Motor control and coordination (governed by kinaesthetic sense and proprioception) can be influenced by many factors, some of which are intrinsic of nature, whereas others are extrinsic (Goodway et al., 2019). Among others, intrinsic factors include gender (Adamo et al., 2012), handedness (Adamo et al., 2012; Marcori & Okazaki, 2020), genetics (Marcori & Okazaki, 2020), direction of development, reciprocal interweaving between motor coordination and control (in addition to muscle and sensory system function), and readiness to execute a task (Goodway et al., 2019). Some concepts that make up extrinsic factors are socio-economic status (SES), the school environment, bonding between parent and infant, motor skill stimulation opportunities, and lack thereof (Venetsanou & Kambas, 2016). Intrinsic and extrinsic factors of importance for this study include SES, gender, and handedness. A brief background of each factor will be provided.

### Socio-Economic Status

SES is most commonly viewed and measured in accordance with education, income, and occupation; however, health, home ownership, or neighbourhood disadvantage can also be used to classify SES (Cockerham et al., 2014). The South African schooling system ranks schools according to different quintiles, where lower quintile schools (one to three) are classified as schools from “poorer” environments and higher quintile schools (four and five) as schools from “richer” environments (Maistry & Africa, 2020). Environmental factors such as where the child grows up, including play opportunities, are directly linked to SES (Malina et al., 2004). Although many studies have researched the association between SES and motor skills (Africa et al., 2021; De Waal & Pienaar, 2020; Morley et al., 2015; Ogbonnaya & Awuah, 2019; Smith, 2011), no known studies have directly linked SES to proprioceptive kinaesthetic coordination. The need thus arises to explore if children from different SES will have different proprioceptive kinaesthetic coordination.

### Gender

Girls and boys grow and mature differently and develop within their own unique timelines (Goodway et al., 2019). Gender as an intrinsic factor of proprioception and kinaesthesia has evidently not been explored in young children. Findings on gender differences in 10- to 14-year-old children showed no significant differences in upper limb proprioception and kinaesthesia between boys and girls (Neagu & Gliga, 2016). Research on adults, however, indicates that gender differences do occur, with Adamo and co-workers (2012) reporting proprioceptive sensorimotor differences between young adult men and women. These researchers suggest that different cortical structures and processing of information specific to each gender underpin these differences (Adamo et al., 2012). With results on older children and young adults being limited and contradictory, research exploring proprioception and kinaesthetic gender differences in young children is thus needed.

### Handedness

Handedness or hand dominance is seen as neural asymmetry where one limb’s functionality is preferred above the other. It is mainly influenced by epigenetics and early childhood behaviour and is therefore affected by intrinsic factors (genetics), but also by several external environmental factors (Marcori & Okazaki, 2020). Children three to five years old typically demonstrate weak and inconsistent hand dominance tendencies (Sharoun & Bryden, 2014), whereas hand dominance becomes established as children mature (7–10 years old) (Sharoun & Bryden, 2014). A study conducted on 307 10- to 14-year-olds reported that proprioceptive and kinaesthetic control of the dominant upper limb was better developed compared to their non-dominant upper limb (Neagu & Gliga, 2016). In adults, similar findings are reported where authors reason different interhemispheric interactions, as well as morphological and region-specific activation as the basis underpinning dominant and non-dominant hand differences (Adamo et al., 2012). No specific literature could be found reporting differences in handedness of young children with regard to proprioception and kinaesthetic sense, thus identifying an area warranting research.

The above-reported literature evidently states that intact proprioceptive kinaesthetic coordination is of high importance for children’s ability to coordinate and control their motor movements. The review of published findings also clearly indicated that school quintile status, gender, and handedness differences with regard to proprioceptive kinaesthetic coordination have not yet been researched among young South African children. The main focus of this study is therefore to determine differences in proprioceptive kinaesthetic coordination of six-year-old children from different school quintiles, of different genders, and with different handedness, in the Motheo District, Mangaung, South Africa.

## Methods

### Study Design

This study was descriptive and analytical in nature and made use of a quantitative cross-sectional study design.

### Study Population and Sampling

Six-year-old children were included in the study. Ten schools from different quintiles (one-five) in the Motheo District, Mangaung, were recruited using convenient sampling, on recommendation of the biostatistician. One quintile one, one quintile two, four quintile three, three quintile four, and one quintile five school participated in the study. The ratio of different quintile schools involved was substantiated by the purpose to collect data which would, to an extent, be a good representative of the South African population especially in the Motheo District, Mangaung. As recommended by the Biostatistician, 40 consent forms were handed out to grade one learners at each of the schools. If a school had fewer than 40 grade one learners, all grade one learners in that school received informed consent letters.

Consent was received for 222 six-year-old boys and girls from low to high SES schools in the Motheo District, Mangaung. After inclusion and exclusion criteria were applied, the sample size included 193 participants with an average age of 6.46 years (SD, 0.27), of which 97 were boys (50.26%) and 96 were girls (49.74%). 20 (10.36%) participants were left-handed and 173 (89.64%) were right-handed. Participants per school quintile included: 32 participants from quintile one schools, eight participants from quintile two schools, 63 participants from quintile three schools, 79 participants from quintile four schools, and 11 participants from quintile five schools. The inclusion criteria allowed for inclusion of six-year-old children from the identified schools, and only if principal and parental consent, as well as child assent, were obtained. Children with incomplete assent and/or consent forms, indicated physical and/or mental disabilities, ear infection, or known vestibular problems as indicated by the teacher, as well as absence on the day of testing, were excluded.

### Procedure

After obtaining ethical clearance from the Health Sciences Research Ethics Committee (HSREC) of the University of the Free State (UFS) (UFS-HSD2020/0143/210), the Department of Basic Education (DoBE), the principals of each school, informed consent from the parents of the recruited children, as well as child assent, a pilot study was conducted. The pilot study included two participants at each of the identified schools to determine if the testing environment was favourable, if trained interpreters were needed, and if the test form was sufficient to ensure good quality data collection. If all above-mentioned aspects were in order during the pilot study, the data was included in the results of the main study.

Data was collected using the proprioceptive kinaesthetic coordination tasks as described in detail under measurement instruments. Testing of proprioceptive kinaesthetic coordination was conducted by the researchers and 14 pre-trained field workers. Only three to four field workers were used at a time at each school. Field workers were trained with a theoretical session (in-class) for an hour where the task items as well as theory on proprioception were discussed. They also had an hour practical session on the task items. All training was presented by the main researcher. Where necessary, the researchers and field workers were assisted by a pre-trained interpreter. The interpreter also followed the same training regime as the field workers. The task items were performed in a randomised sequence in order to reduce the possible occurrence of fatigue and to prevent participants being able to communicate the order of the items to their peers. Testing commenced in the morning at a time that was suitable for each school and at a time that did not hinder academic performance, and continued for one and a half to two hours in order to test the whole participant group at the relevant school.

### Ethical Considerations

This study was approved by the (HSREC) of the (UFS) (UFS-HSD2020/0143/210) as well as the DoBE. All aspects of data collection were conducted according to the ethical guidelines and principles of the South African Guidelines for Good Clinical Practice and the Medical Research Council (MRC) Ethical Guidelines for Research. Consent from the parents/guardians and assent from the participants were obtained prior to commencement of the data collection. Instead of their name, a participant number was used to ensure privacy and all data was handled confidentially according to the above-mentioned guidelines. Measurement errors were reduced as far as possible by ensuring adequate training of the field workers and the interpreter, as well as proper demonstration and explanation of the task-items to participants. Optimal testing time (length and time of day) was allocated when data was collected, and handling of raw data was quality controlled by the main researcher and kept safe in a secure location and then in a password protected Excel spreadsheet.

### Measurement Instruments

A series of five task items was identified and used to measure the proprioceptive and/or kinaesthetic coordination abilities of the participants involved (Cheatum & Hammond, 2000; Chu, 2017). Some of these tasks (Angels-in-the-Snow, Rhomberg, and Finger-to-Nose tests) have been used by other researchers to determine proprioceptive abilities in terms of motor control, motor coordination, and kinaesthesia (Moran et al., 2005; Swaine et al., 2005). Although these tasks have no reported validity or reliability (Cheatum & Hammond, 2000; Chu, 2017), they do have set execution instructions which were used.

A test form was developed for each participant, which included: sequence number; participant’s gender, height, weight, birth date and chronological age, and a table to record raw scores. Formal administration of the task items took approximately 10–15 minutes per participant but was dependent on the degree of difficulty experienced by the participant. Handedness was determined on their test form by indicating the preferred hand that participants used to fill in their assent form. The preferred arm chosen by each participant when executing a task was also regarded as the dominant side. As handedness refers to the preferred usage of one limb over the other, it will be used as a collective term for preferred arm or dominant hand within the results and discussion sections.

Task items and guidelines for the execution of the items included:

- *Angels-in-the-Snow task*
  Angels-in-the-Snow is used to evaluate children’s coordination (Mutti et al., 2017). In the current study, the researchers taped a solid straight line on a yoga mat for the participant to lie on, with the line being in the middle of their bodies. Researchers told the participants that they would point to limb/s that need to slide over the floor, reminded them that they should not lift their limb/s off the ground, and that they need to return it to the normal relaxed position. Participants needed to perform a series of eleven consecutive movements (Cheatum & Hammond, 2000) with a maximum repetition of four per movement.
- *Rhomberg task*
  This task evaluated balance in a standing position (Cheatum & Hammond, 2000; Henriques et al., 2014). Participants stood feet together, toes facing forward, with arms relaxed at their side. The examiner asked the participant to stand up straight and keep balance with their eyes closed. Data was recorded as the number of seconds the participant was able to stand in the specified position, with 30 secs being the maximum.
- *Finger-to-Nose task*
  Different versions of the Finger-to-Nose task exist (Sayar & Űnűbol, 2017; Swaine et al., 2005). However, for the purpose of this study, the Finger-to-Nose task was done by extending both arms next to the body at shoulder level and then with each index finger touching the tip of the nose and returning the arm to the original position (alternating the left and right arms). Participants executed the task with their eyes closed. The required number of successful repetitions performed by the participant was three (per side), indicating a maximum recorded score of six.
- *Shoulder-level-arm-raise task*
  This item assessed movement of a limb around a joint and in space and was executed as set out by Cheatum and Hammond (2000). Participants stood with eyes closed and then raised arm/s to the front with the following movements being performed: raising preferred arm to shoulder level, then non-preferred arm, and then both arms. The required number of successful repetitions performed by the participant was four arm lifts for the preferred arm, non-preferred arm and both arms.
- *Force perception Task*
  This task item was set out and explained by Chu (2017) and was used to assess force sense. The participants stood with arms stretched out in front of them with their eyes closed. A light weighted object (500 g) was placed in one hand and a heavier weighted object (1 kg) was placed in the participant’s other hand. The participant then needed to tell the researcher which hand held the heavier object. This task was performed only once.

## Data Analysis

Collected data were entered into a Microsoft Excel spreadsheet, converted into a SAS data set, and analysed by a Biostatistician using the SAS software (SAS Institute Inc, 2017).

### Descriptive Statistics

Quantitative variables were summarised using descriptive statistics (mean, SD) overall, for the whole sample, by school quintile, gender, and handedness. Descriptive statistics also indicated whether participants fell below, within, or above the proprioceptive kinaesthetic coordination norms, as established in the master’s dissertation of Bonafede (2021).

To ensure that the inclusion of all task items was deemed necessary in the analysis, Pearson correlation coefficients analysed the relationship between the variables of the five task items. Correlations (*r*) were interpreted as follows:* r* < 0.3 was regarded as a low correlation, with *r* > 0.7 indicating variables that correlated highly with one another (Schober et al., 2018). As seen in Table 2, correlations between task items are quite low (*r* < 0.3), which suggests that various tasks do indeed measure different aspects of proprioceptive kinaesthetic coordination. High correlations (*r* > 0.7) were only the case between variables of the same task item, which was executed with different limbs. Consequently, all variables of the five task items were included in further analyses (Table 1).

**Table 1:** Norms for the five task items. *Source:* Bonafede (2021)

	Angels-in-the-Snow	Rhomberg	Finger-to-Nose	Shoulder-level-arm-raise	Force perception
Theoretical Min Score	0	0 s	0 per hand	0 per arm	Incorrect hand identified
Theoretical Max Score	11	30 s	3 per hand	4 per arm	Correct hand identified
Norm	6–10 reps	22–30 s	2–3 reps per arm	2–4 reps (dominant arm & both arms) 1–4 reps (non-dominant arm)	Correct identification of the heavier object

*Min:* minimum, *Max:* maximum, *reps*: repetitions

**Table 2:** Pearson correlations coefficients for the five task items *Source:* no source

Task items	Angels	Rhomberg	F-to-N (*L*)	F-to-N (*R*)	S-level (*D*)	S-level (*n*-*D*)	S-level (both)	Force-*P*
Angels	1.00	0.17	0.31	0.27	0.16	0.18	0.15	0.08
Rhomberg		1.00	0.09	0.11	0.19	0.14	0.29	− 0.03
Finger-to-Nose (*L*)		1.00	0.79*	0.09	0.11	0.05	0.08	
Finger-to-Nose (*R*)			1.00	0.10	0.11	0.01	0.09	
Shoulder-level (*D*)				1.00	0.74*	0.65	0.06	
Shoulder-level (*n*-*D*)					1.00	0.70*	0.09	
Shoulder-level (both)						1.00	0.11	
Force Perception							1.00	

Angels: Angels-in-the-Snow, F-to-N: Finger-to-Nose, *L*: left index finger, *R*: right index finger, S-level/shoulder level: shoulder-level-arm-raise, *D*: dominant arm, *n*-*D*: non-dominant arm, both: both arms, Force-P: force perception

*:* r* > 0.7

### Comparison of School Quintiles, Genders and Handedness

Quantitative data were compared between genders and with regard to handedness using the nonparametric Wilcoxon two-sample test (null hypothesis: distribution of the two populations compared is the same). The school quintiles were compared with respect to quantitative data using the Cochran–Mantel–Haenszel (correlation) Chi-square test (null hypothesis: zero correlation between variable analysed and school quintile), and the binary variable. Force perception was compared using Fisher’s exact test (null hypothesis: independence between variable force perception and the variables gender/handedness). In each case, the associated P-value is reported with *p* < 0.05 indicating statistical significance. Cohen’s *d* effect sizes were used to indicate practical significance for handedness and gender differences in the Angels-in-the-Snow, Rhomberg, and Finger-to-Nose tasks. A value of 0.2 indicated a small practical effect, 0.5 a medium practical effect, and 0.8 a large practical effect (Salkind, 2010).

## Results

Table 3 summarises the Angels-in-the-Snow task results. Results show that participants from different quintile schools (one-five) performed similarly, with the exception of quintile three schools. They had a lower mean value (6.37) compared to the other quintile schools’ mean values, which are all higher than 8.00. Differences between school quintiles were, however, not statistically or practically significant. No significant or practical differences were found between girls and boys, as well as between left-handed and right-handed participants.

**Table 3:** Angels-in-the-snow *Source:* no source

	Group	Mean	SD	*P*-value	*Cohen’s d*	Below		Within		Above	
						*n*	%	*n*	%	*n*	%
School quintile	1	8.50	2.53	0.9693		3	9.38	22	68.75	7	21.88
	2	8.50	2.07			1	12.50	6	75.00	1	12.50
	3	6.37	3.42			25	39.68	27	42.86	11	17.46
	4	8.10	2.38			11	13.92	55	69.62	13	16.46
	5	8.91	1.58			0	0.00	10	90.91	1	9.09
Gender	Girls	7.76	3.02	0.3792	0.0672	19	19.79	59	61.46	18	18.75
	Boys	7.57	2.73			21	21.65	61	62.89	15	15.46
Handedness	Left	7.30	2.66	0.3899	0.1408	4	20.00	13	65.00	3	15.00
	Right	7.71	2.90			36	20.81	107	61.85	30	17.34

SD: standard deviation, below/within/above: indicating if value was below/within/above the set norms, *n*: number of participants, %: percentage

*p < 0.05; ^#^: *d* > 0.2, ^##^: *d* > 0.5, ^###^:* d* > 0.8

For the Rhomberg task, as depicted in Table 4, the participants from the quintile one school performed the best on average (mean = 29.19) and the participants from the quintile five school performed second best (mean = 28.36). No significant differences were, however, observed between participants from different quintile schools. There were also no statistically or practically significant differences between genders in this task. Handedness did not reflect in the results of this task as it was not relevant to the Rhomberg task with hands being kept relaxed next to the participant’s body.

**Table 4:** Rhomberg *Source:* no source

	Group	Mean	SD	*P*-value	*Cohen’s d*	Below		Within	
						*n*	%	*n*	%
School quintile	1	29.19	7.55	0.5766		7	21.88	25	78.13
	2	23.75	9.22			3	37.50	5	62.50
	3	25.89	7.49			14	22.22	49	77.78
	4	25.14	8.07			22	27.85	57	72.15
	5	28.36	3.67			1	9.09	10	90.91
Gender	Girls	26.00	6.09	0.7621	0.1253	21	21.88	75	78.13
	Boys	25.04	8.31			26	26.80	71	73.20

SD: standard deviation*, *below/within: indicating if value was below/within the set norms, *n*: number of participants, %: percentage

*p < 0.05; ^#^: *d* > 0.2, ^##^: *d* > 0.5, ^###^:* d* > 0.8

Table 5 reflects results of the Finger-to-Nose task. Differences between participants of different quintile school were not statistically significant. There were also no statistically or practically significant differences between genders, as boys and girls performed very similarly when both the left and right index fingers touched the nose. When touching the nose with the left index finger, no statistically significant differences between left- and right-handed participants were observed. However, a practically significant effect of a small size (*d* = 0.3460) is seen favouring right-handed participants when touching the nose with the left index finger. A statistically significant difference (*p* = 0.0125) with a small practical effect (*d* = 0.3882) is reported when touching the nose with the right index finger, as right-handed participants performed better.

**Table 5:** Finger-to-Nose *Source:* no source

	Group	Mean	SD	*P*-value	*Cohen’s d*	Below		Within	
						*n*	%	*n*	%
*Left index finger*									
School quintile	1	2.25	1.08	0.4091		7	21.88	25	78.13
	2	1.88	1.55			3	37.50	5	62.50
	3	2.29	1.14			16	25.40	47	74.60
	4	2.54	0.87			12	15.19	67	84.81
	5	1.82	1.33			4	36.36	7	63.64
Gender	Girls	2.33	1.04	0.8106	0.0160	23	23.96	73	76.04
	Boys	2.35	1.10			19	19.59	78	80.41
Handedness	Left	2.15	1.23	0.3998	0.3460^#^	5	25.00	15	75.00
	Right	2.36	1.05			37	21.39	136	78.61
*Right index finger*									
School quintile	1	2.44	0.95	0.5996		4	12.50	28	87.50
	2	1.88	1.55			3	37.50	5	62.50
	3	2.48	1.01			12	19.05	51	80.95
	4	2.65	0.73			8	10.13	71	89.87
	5	1.91	1.30			3	27.27	8	72.73
Gender	Girls	2.53	0.87	0.9034	0.1024	12	12.50	84	87.50
	Boys	2.43	1.04			18	18.56	79	81.44
Handedness	Left	2.15	0.99	0.0125*	0.3882^#^	4	20.00	16	80.00
	Right	2.52	0.95			26	15.03	147	84.97

SD: standard deviation, below/within/above indicating if value was below/within/above the set norms, *n*: number of participants, %: percentage

*p < 0.05; ^#^: *d* > 0.2, ^##^: *d* > 0.5, ^###^: *d* > 0.8

The results for shoulder-level-arm-raises are depicted in Table 6. Data are presented for this task when executed with the dominant arm, non-dominant arm, and both arms. No statistical differences were observed between participants from different quintile schools, whether the shoulder-level-arm-raise was executed with the dominant arm, non-dominant arm, or both arms. A statistically significant difference (*p* = 0.0288) with a small practical effect (*d* = 0.3139) was found between genders when executing the task with the dominant arm, where boys performed better than girls. No statistical differences occurred between boys and girls when using their non-dominant arm or both arms to execute the task. Handedness seemed not to influence task execution as no practical or statistically significant differences were evident between left-handed and right-handed participants when executing all three movements.

**Table 6:** Shoulder-level-arm-raise *Source:* no source

	Group	Mean	SD	*P*-value	*Cohen’s d*	Below		Within	
						*n*	%	*n*	%
*Dominant arm*									
School quintile	1	2.72	1.57	0.3616		7	21.88	25	78.13
	2	2.75	1.83			2	25.00	6	75.00
	3	2.83	1.59			14	22.22	49	77.78
	4	3.08	1.51			15	18.99	64	81.01
	5	2.73	1.85			3	27.27	8	72.73
Gender	Girls	2.66	1.67	0.0288*	− 0.3139^#^	26	27.08	70	72.92
	Boys	3.14	1.44			15	15.46	82	84.54
Handedness	Left	3.05	1.50	0.6650		3	15.00	17	85.00
	Right	2.88	1.58			38	21.97	135	78.03
*Non-dominant arm*									
School quintile	1	2.56	1.58	0.5382		7	21.88	25	78.13
	2	2.13	2.03			3	37.50	5	62.50
	3	2.60	1.66			14	22.22	49	77.78
	4	2.84	1.56			14	17.72	65	82.28
	5	2.27	2.00			4	36.36	7	63.64
Gender	Girls	2.55	1.66	0.2990	− 0.1225	23	23.96	73	76.04
	Boys	2.75	1.61			19	19.59	78	80.41
Handedness	Left	3.10	2.60	0.4377		2	10.00	18	90.00
	Right	1.25	1.67			40	23.12	133	76.88
Both arms									
School quintile	1	2.78	1.54	0.3486		7	21.88	25	78.13
	2	2.25	1.98			3	37.50	5	62.50
	3	2.86	1.65			17	26.98	46	73.02
	4	2.97	1.48			15	18.99	64	81.01
	5	3.09	1.58			2	18.18	9	81.82
Gender	Girls	2.90	1.55	0.8207	0.0189	20	20.83	76	79.17
	Boys	2.87	1.60			24	24.74	73	75.26
Handedness	Left	3.05	1.32	0.9362		3	15.00	17	85.00
	Right	2.86	1.60			41	23.70	132	76.30

SD: standard deviation, below/within/above: indicating if value was below/within/above the set norms, *n*: number of participants, %: percentage

*p < 0.05; ^#^: *d* > 0.2, ^##^:* d* > 0.5, ^###^: *d* > 0.8

Results for the force perception task are presented in Table 7. There were no statistically significant differences regarding school quintiles or handedness. A statistically significant difference (*p* = 0.0322) between boys and girls was observed, where the girls outperformed the boys. There was no statistically significant difference between left-handed and right-handed participants when executing this task.

**Table 7:** Force perception *Source:* no source

	Group	%	*P*-value	Below		Within	
				*n*	%	*n*	%
School quintile	1	75.0	0.0993	8	25.00	24	75.00
	2	62.5		3	37.50	5	62.50
	3	85.7		9	14.29	54	85.71
	4	87.3		10	12.66	69	87.34
	5	81.8		2	18.18	9	81.82
Gender	Girls	89.6	0.0322*	10	10.42	86	89.58
	Boys	77.3		22	22.68	75	77.32
Handedness	Left	80.0	0.7499	4	20.00	16	80.00
	Right	83.8		28	16.18	145	83.82

SD: standard deviation, below/within: indicating if value was below/within the set norms, *n*: number of participants, %: percentage

*p < 0.05

## Discussion

The aim of this study was to determine differences in proprioceptive kinaesthetic coordination of 6-year-old children from different school quintiles in South Africa, of different genders, and with different handedness.

### School Quintile Differences

Findings of this study report no statistical or practical significant differences in the proprioceptive kinaesthetic coordination of participants from different quintile schools and SES backgrounds. Studies exploring children’s proprioceptive kinaesthetic coordination between various SES environments exclusively are scarce, and the results of the current study could therefore not be discussed at the hand of other similar studies. Literature is, however, clear on gross motor skill differences between children from different SES settings (Balli, 2016; Draper et al., 2012; Hilpert et al., 2017; Okely et al., 2004; Tomaz et al., 2019), with findings mostly favouring children from higher SES, with the exception of static balancing tasks such as the Rhomberg (Fjørtoft, 2004; Pienaar & Kemp, 2014). Although proprioception and kinaesthetic sense govern gross motor movements explored in these studies (unilateral movements, bilateral coordination, and static balancing), the results are not comparable and no relation can be drawn with our findings, as they did not exclusively explore proprioception or kinaesthetic sense.

### Gender Differences

Research states that minor gender differences exist in performance of motor movements before the growth spurt and onset of adulthood (Malina et al., 2004). Minimal gender differences in respect to six-year-old children’s proprioceptive kinaesthetic coordination were also observed in this study, with only two task items indicating significant differences. This was observed in the shoulder-level-arm-raise task when the dominant arm was used, where boys outperformed girls, and in the force perception task, where the girls outperformed the boys. Minimal significant differences found in the current study can be due to ongoing proprioceptive kinaesthetic coordination development at the age of six since the proprioceptive system is only matured later (Úbeda-Pastor et al., 2018). As with other developmental domains, minimal gender differences exist between boys and girls at this age (Goodway et al., 2019; Neagu & Gliga, 2016).

In South Africa, a study conducted on 160 participants aged six to twelve years reported no significant differences in terms of motor movements between genders (Pila-Nemutandani et al., 2020). Although the mentioned study did not exclusively evaluate proprioception or kinaesthetic sense, it is still of value as it links motor movements and gender. In a study done on 84 four- to eight-year-old children using motor sequencing items and bilateral coordination tasks, no differences in bilateral coordination were observed between genders (Cardoso & Magalhāes, 2009). When five-year-old children were evaluated with the BOT-2 short form, results for bilateral coordination (jumping in place, with the same side synchronised) showed no statistically significant differences between boys and girls (Matarma et al., 2020). Although not completely equivalent, our results to some extent support the fact that when children move in a coordinated manner, gender differences are minimal at a young age.

### Handedness Differences

No significant differences were observed between right-hand and left-hand dominant children during execution of the Angels-in-the-Snow, Rhomberg, shoulder-level-arm-raise, and force perception tasks. However, for the Finger-to-Nose task, a statistically significant difference was noted between left- and right-handed children where right-handed children performed better when touching the right index finger to their nose. The study of Triggs and co-workers (2000) concluded that the type of task performed is influenced by hand dominance. The study included 60 participants, of which 30 were left-handed and 30 were right-handed. In tasks that require usage of the right hand, right-handed participants performed better with their dominant hand than non-dominant hand, and vice versa (Triggs et al., 2000). This was congruent with our findings during the Finger-to-Nose task, but only for right-handed participants. Poorer performance by the left-handed children in our study can be explained by the pressure the environment places on left-handers to sometimes use their right hand to perform tasks or use right-handed equipment. This causes discord between intrinsic dynamics, leading them to have less prominent asymmetrical behaviours compared to right-handed individuals (Marcori & Okazaki, 2020).

## Conclusion

The proprioceptive kinaesthetic coordination of six-year-olds from South Africa from different school quintiles did not significantly differ. Gender differences favoured girls in terms of force perception, while boys portrayed better performance in the shoulder-level-arm-raise task when executed with the dominant arm. Right-handed six-year-olds showcased significantly better Finger-to-Nose movements than left-handed children, when touching the nose with their right index finger.

## Recommendations and Limitations

Although the five task items are confirmed to measure different aspects of proprioceptive kinaesthetic coordination (see Table 2), we recommend that future research determine the reliability and validity values of the task items to ensure quality of results. Practical implications of the findings indicate that proprioceptive kinaesthetic coordination interventions do not need to be customised for children from different SES backgrounds. Although proprioceptive kinaesthetic coordination of boys and left-handed children might need a bit more attention, it is recommended that sufficient movement opportunities should be offered to six-year-old children portrays proprioceptive kinaesthetic coordination difficulties. Increasing muscular strength and perception of force application can be achieved by playing on apparatus such as jungle gyms or manipulating objects of different weights. Movement opportunities relating to spatial awareness, such as obstacle courses (climbing through tires, jumping over bricks, etc.), are also of importance and can improve young children’s overall proprioception and kinaesthetic sense. Adequate development and performance of these proprioceptive kinaesthetic coordination skills set children’s motor skill development on a positive trajectory, thus being of utmost importance for success in other developmental domains.

The first limitation of this study was a low response rate regarding the receipt of consent forms. Although 400 consent forms were handed out, only 222 were received back. This low response rate was, to a large extent, due to the Covid-19 pandemic which forced schools to work on a rotational attendance schedule and resulted in some children not being able to receive, or to bring back consent forms on time. Another limitation in the study was that some of the school quintile groupings only included one school per quintile (one, two, five), whereas other quintile groupings had more than one school representing that quintile status. However, the ratio of quintiles within the study is still seen as a good representative of the South African population as derived from the Motheo District, Mangaung. Lastly, only handedness was observed where the role of leg dominance could also have played a role in proprioceptive kinaesthetic coordination of these six-year-old children.

Although a few limitations exist, the findings of the current study can also be seen in a positive light, as the topic is novel within the research field of young developing children. With proprioception and kinaesthetic sense being a foundational aspect to children’s movement success, our findings can open the door for future research to further enhance our understanding of proprioceptive kinaesthetic coordination in young children.
